# NrtR Regulates the Type III Secretion System Through cAMP/Vfr Pathway in *Pseudomonas aeruginosa*

**DOI:** 10.3389/fmicb.2019.00085

**Published:** 2019-01-30

**Authors:** Yongxin Jin, Mengjing Zhang, Feng Zhu, Qianqian Peng, Yuding Weng, Qiang Zhao, Chang Liu, Fang Bai, Zhihui Cheng, Shouguang Jin, Weihui Wu

**Affiliations:** ^1^State Key Laboratory of Medicinal Chemical Biology, Key Laboratory of Molecular Microbiology and Technology of the Ministry of Education, Department of Microbiology, College of Life Sciences, Nankai University, Tianjin, China; ^2^College of Life Sciences, Nankai University, Tianjin, China; ^3^Department of Molecular Genetics and Microbiology, College of Medicine, University of Florida, Gainesville, FL, United States

**Keywords:** *Pseudomonas aeruginosa*, NrtR, cAMP, CyaB, type III secretion system

## Abstract

The type III secretion system (T3SS) plays an important role in the pathogenesis of *Pseudomonas aeruginosa*. Expression of the T3SS is controlled under a complicate regulatory network. In this study, we demonstrate that NrtR (PA4916) is involved in the T3SS expression and pathogenesis of *P. aeruginosa* in a mouse acute pneumonia model. Overexpression of the T3SS central activator ExsA or exogenous supplementation of cAMP restored the expression of T3SS in the Δ*nrtR* mutant, suggesting that NrtR might regulate T3SS through the cAMP-Vfr signaling pathway. Further experiments demonstrated that the decrease of cAMP content is not due to the expression change of adenylate cyclases or phosphodiesterase in the Δ*nrtR* mutant. As it has been shown that *nadD2* is upregulated in the Δ*nrtR* mutant, we overexpressed *nadD2* in wild type PAK, which reduced the intracellular cAMP level and the expression of the T3SS genes. Meanwhile, deletion of *nadD2* in the Δ*nrtR* mutant restored the expression and secretion of the T3SS. Co-immunoprecipitation assay revealed an interaction between NadD2 and the catalytic domain of the adenylate cyclase CyaB. Further *in vitro* assay indicated that NadD2 repressed the enzymatic activity of CyaB. Therefore, we have identified a novel regulatory mechanism of T3SS in *P. aeruginosa*.

## Introduction

*Pseudomonas aeruginosa* is a ubiquitous Gram-negative bacterium that can cause both acute and chronic infections in individuals with compromised immunity such as cancer patients and those with cystic fibrosis (Crowe et al., 1982; Sherertz and Sarubbi, 1983; Lyczak et al., 2002; Sousa and Pereira, 2014).

The type III secretion system (T3SS) is an important virulence factor of *P. aeruginosa*, through which effector proteins are directly injected into the cytosols of eukaryotic host cells, inhibiting host defense by inducing cell death in polymorphonuclear phagocytes, macrophages, and epithelial cells (Dacheux et al., 1999; Hauser and Engel, 1999; Kaufman et al., 2000). Expression of the T3SS confers an increased virulence in *P. aeruginosa* and is associated with poor clinical outcomes (El-Solh et al., 2012), whereas strains with defective T3SS display attenuated virulence in mouse acute infection models (Smith et al., 2004). To date, four effector proteins have been identified and well characterized in *P. aeruginosa*, i.e., ExoS, ExoT, ExoU, and ExoY (Hauser, 2009). However, majority of *P. aeruginosa* isolates do not encode all of the four effectors (Feltman et al., 2001). For example, strain PAK expresses ExoS, ExoT, and ExoY, while strain PA14 expresses ExoU, ExoT, and ExoY.

In *P. aeruginosa*, the T3SS is induced in response to a variety of environmental conditions, such as direct contact with host cell, calcium depletion and the presence of serum (Kim et al., 2005; Hayes et al., 2010). The expression of T3SS is activated by ExsA, an AraC-type DNA binding protein, which recognizes and binds to two adjacent highly conserved consensus sequences in the promoter region of the T3SS genes (Hovey and Frank, 1995; Brutinel et al., 2008). The ExsA activity and transcriptional regulation on T3SS are intimately coupled to secretion by a partner-switching model involving three other proteins: ExsC, ExsD, and ExsE (Brutinel and Yahr, 2008). Under non-inducing condition, the secretable repressor ExsE is kept inside bacterial cytosol and binds to ExsC, and ExsD binds to and inactivates ExsA. Whereas under inducing environment, ExsE is secreted by the T3SS machinery, which releases ExsC to sequester its low affinity partner ExsD, resulting in free ExsA that activates the transcription of whole T3SS gene cluster (McCaw et al., 2002; Dasgupta et al., 2004; Rietsch et al., 2005; Urbanowski et al., 2005, 2007; Thibault et al., 2009; Diaz et al., 2011).

The virulence factor regulator Vfr is a cAMP-dependent transcriptional regulator. It was originally identified as an activator of extracellular protease and exotoxin A expression. Now it is appreciated as a global regulator of virulence gene expression, including T3SS and pili biosynthesis genes (West et al., 1994; Wolfgang et al., 2003; Marsden et al., 2016). Intracellular cAMP is generated by adenylate cyclases CyaA and CyaB and hydrolyzed by the phosphodiesterase CpdA in *P. aeruginosa* (Wolfgang et al., 2003; Fuchs et al., 2010). Besides, other regulators and proteins are also known to affect expression of the T3SS, such as the *rhl* quorum sensing system (Hogardt et al., 2004), stationary-phase sigma factor RpoS (Hogardt et al., 2004), transcriptional activator PsrA (Shen et al., 2006), global regulator MexT (Jin et al., 2011), alginate biosynthesis protein MucA (Wu et al., 2004), RNA-binding proteins RsmA and Crc (Mulcahy et al., 2006; Dong et al., 2013), small proteins PtrA, PtrB, and PtrC (Ha et al., 2004; Wu and Jin, 2005; Jin et al., 2011), tryptophan synthase TrpA (Shen et al., 2008), tryptophan dioxygenase KynA (Shen et al., 2008), pseudouridinase enzyme TruA (Ahn et al., 2004), nitrite reductase NirS (Van Alst et al., 2009), magnesium transporter MgtE (Anderson et al., 2010), two-component system AlgZR (Intile et al., 2014), DNA binding protein Fis (Deng et al., 2017), multiple virulence regulator SuhB and RNA helicase DeaD (Li et al., 2013; Intile et al., 2015). However, the molecular mechanism by which most of these proteins regulate T3SS is not fully elucidated yet. All of these suggest that expression of T3SS is controlled under a complicate regulatory network. Therefore, we aimed to identify novel regulators of T3SS and their regulatory mechanisms.

In the present study, we identified that NrtR (PA4916) is required for the expression of T3SS in *P. aeruginosa*. *In vivo* studies suggest that NrtR plays an important role in the pathogenesis of *P. aeruginosa*. Further studies demonstrated that NrtR affects expression of the T3SS through cAMP/Vfr signaling system that lies upstream of the ExsA. We demonstrated that NadD2 is involved in the NrtR mediated regulation of the T3SS by inhibiting the CyaB enzymatic activity and subsequent reducing intracellular cAMP level.

## Materials and Methods

### Ethics Statement

All animal studies complied with National and Nankai University guidelines regarding the use of animals in research. All animal experiment protocols have been approved by the institutional animal care and use committee of the College of Life Sciences of Nankai University (permit number NK-04-2012).

### Bacterial Strains, Plasmids, and Growth Conditions

Bacterial strains and plasmids used in this study are listed in Table 1. Both *E. coli* and *P. aeruginosa* were grown in Luria-Bertani broth (LB) medium (Deng et al., 2017) or on LB agar (Deng et al., 2017) plates at 37°C. Whenever needed, antibiotics were used at following concentrations (μg/ml): for *E. coli*, ampicillin 100, tetracycline 10, spectinomycin 50, streptomycin 25, gentamicin 10; for *P. aeruginosa*, carbenicillin 150, tetracycline 50, spectinomycin 200, streptomycin 200, gentamicin 100. When needed, 1 mM IPTG (isopropyl β-D-1-thiogalactopyranoside) or 50 mM cAMP were added to culture medium. Primers used to make various constructs and in RT-qPCR are listed in Table 2.

**Table 1:** Bacterial strains and plasmids used in this study.

Strain or plasmid	Description	Source
***E. coli* strains**		
DH5α	F^-^ ϕ 80d*lacZ*ΔM15 *endA1 recA1 hsdR17*(r_K_^-^ m_K_^+^) *supE44 thi-1 relA1* Δ(*lacZYA-argF*)*U169 gyrA96 deoR*	TransGen
S17-1	RP4-2 Tc::Mu Km::Tn*7* Tp^r^ Sm^r^ Pro Res^-^ Mod^+^	Dr. Ramphal
HB101	Source for wild type *lac*P1 promoter sequence	TransGen
***P. aeruginosa* strains**		
PA14	Wild type *P. aeruginosa* strain	Liberati et al., 2006
PAK	Wild type *P. aeruginosa* strain	David Bradley
*exsA*::Tn	PA14 with *exsA* disrupted by insertion of Tn	Liberati et al., 2006
PA4336::Tn	PA14 with PA4336 inserted with Tn	Liberati et al., 2006
PA4916::Tn	PA14 with PA4916 inserted with Tn	Liberati et al., 2006
PA0020::Tn	PA14 with PA0020 inserted with Tn	Liberati et al., 2006
PA4753::Tn	PA14 with PA4753 inserted with Tn	Liberati et al., 2006
*exsA*::Ω	PAK with *exsA* disrupted by insertion of cassette Ω; Sp^r^, Sm^r^	Li et al., 2013
Δ*nadD2*	PAK with *nadD2* deleted	This study
Δ*nadD2-nrtR*	PAK with *nadD2-nrtR* operon deleted	This study
ΔPA4916	PAK with PA4916 deleted	This study
ΔPA4916*/att7*::PA4916	ΔPA4916 with PA4916 inserted into the chromosome with mini-Tn7 insertion	This study
Δ*vfr*	PAK with *vfr* gene deleted	This study
Δ*nrtR*Δ*vfr*	PAK with both *nrtR* and *vfr* gene deleted	This study
**Plasmids**		
pDN19	Shuttle vector between *E. coli* and *P. aeruginosa*; Tc^r^	Li et al., 2013
pMMB67EH	Shuttle vector between *E. coli* and *P. aeruginosa*; Amp^r^	Li et al., 2016
pE1553	Promoterless pUCP20; Amp^r^	Li et al., 2016
pE1553a	*cyaA*-flag with own promoter in pE1553; Amp^r^	This study
pE1553b	*cyaB*-flag with own promoter in pE1553; Amp^r^	This study
pE1553-*cpdA*	*cpdA*-flag with own promoter in pE1553; Amp^r^	This study
pMMB67-*cyaA*	*cyaA*-flag cloned into pMMB67EH driven by tac promoter; Amp^r^	This study
pMMB67-*cyaB*	*cyaB*-flag cloned into pMMB67EH driven by tac promoter; Amp^r^	This study
pMMB67-*nadD2*	*nadD2* gene of PAK on pMMB67EH driven by tac promoter; Amp^r^	This study
pMMB67-4918-20	PA4918-20 gene of PAK on pMMB67EH driven by tac promoter; Amp^r^	This study
pMMB67EH-*cyaB*_217-463_-His	CyaB_217-463_-His cloned into pMMB67EH driven by tac promoter; Amp^r^	This study
pMMB67EH-*cyaA*-His	CyaA-His cloned into pMMB67EH driven by tac promoter; Amp^r^	This study
pUCP24	Shuttle vector between *E. coli* and *P. aeruginosa*; Gm^r^	Shi et al., 2015
pUCP24-*nadD2*-Flag	*nadD2*-Flag cloned into pUCP24; Gm^r^	This study
		
*exsA*	*exsA* gene of PAK on pDN19 driven by *lac* promoter; Tc^r^	Li et al., 2013
*lac*P1	*Lac*P1 promoter of *E. coli* fused to promoterless *lacZ* on pDN19*lacZ*Ω; Sp^r^, Sm^r^, Tc^r^	This study
pUC18T-mini-Tn7T-Gm	mini-Tn7 base vector insertion into chromosome attTn7 site, Gm^r^	Li et al., 2013
pUC18T-mini-Tn7T-P*_exsC_*-*exsCEBA*-Flag-*ExsD*	*exsCEBAD* gene with *exsA*-Flag tagged on pUC18T-mini-Tn7T driven by *exsC* promoter, Gm^r^	This study
pTNS3	Helper plasmid encoding Tn7 site-specific transposition pathway; Amp^r^	Deng et al., 2017
pEX18Tc	Gene knockout vector; Tc^r^	Deng et al., 2017
pZF01	PA4916 gene deletion on pEX18Tc; Tc^r^	This study
pEX18Tc-Δ*nadD2*	*nadD2* gene deletion on pEX18Tc; Tc^r^	This study
pEX18Tc-Δ*nadD2-nrtR*	*nadD2-nrtR* operon deletion on pEX18Tc; Tc^r^	This study
pEX18Tc-Δ*vfr*	*vfr* gene deletion on pEX18Tc; Tc^r^	This study
pEX18Tc-Δ*cyaA*	*cyaA* gene deletion on pEX18Tc; Tc^r^	This study
pEX18Tc-Δ*cyaB*	*cyaB* gene deletion on pEX18Tc; Tc^r^	This study
pZF02	PA4916 gene on pUC18T-mini-Tn7T-Tc; Tc^r^	This study
pET16b	Expression vector, Kan^r^	Novagen
pET16b-*nadD2*	*nadD2* gene of PAK cloned into pET16b	This study
pET28a	Expression vector, Kan^r^	Novagen
pET28a-*cyaB*_217-463_	*cyaB* gene encoding amino acid positions 217–463 cloned into pET28a	This study

**Table 2:** Primers used in this study.

Primer	Sequence 5^′^–3^′b^	Source
PA4916UF^a^	CTCGGAATTCTTCCAGACGAAGAAGTCGTAG	This study
PA4916UR	CTGCTCTAGACGTCACTCCTCTCTTCAGCCC	This study
PA4916DF	GAGCTCTAGACCTGCCGCGCTTGCTAGACG	This study
PA4916DR	GCCCAAGCTTAAATCATCGAGTCGCTGGTCCCC	This study
*nadD2*UF	GGAATTCGAAGACCTCCACCTCCAGTGTCG	This study
*nadD2*UR	GCTCTAGAAGAGAGGAGTGACGATGAGTTCAG	This study
*nadD2*DF	GCTCTAGAAGAAAATACCTTCCACTGCG	This study
*nadD2*DR	CCCAAGCTTGAGCAGGTTCTGCACAATGC	This study
*cyaA*UF	GGAATTCCGGCATCCGTTGTTCCGCGCGGAGATCCAG	This study
*cyaA*UR	GCTCTAGAGGGCGTCCGGGCACAGGCAAGGCCAGGCG	This study
*cyaA*DF	GCTCTAGACCCAGCGCCGCACCGCGCGGGGCTCGAC	This study
*cyaA*DR	CCCAAGCTTCGCCGGCGAAGGCAAGGTCTCGATCCTC	This study
*cyaB*UF	GGAATTCGGAAAGTCAGGTCGGACGCTTCCGCGATG	This study
*cyaB*UR	GCTCTAGAGCGCTGGAGAGGATCCCTGTGTATTTTCG	This study
*cyaB*DF	GCTCTAGAGTTCGTCGAACGCCGCCGGCAGTTCGTCGCGCC	This study
*cyaB*DR	CCCAAGCTTCCGCTCGGCTGGGCCGCGCGGCGCTGGC	This study
*vfr*UF	CTCGGAATTCGTAGCAGATGTCGTAGATGTTG	This study
*vfr*UR	CTGGGGTACCCGAGTCCCGAAAGAATAAAG	This study
*vfr*DF	GAGGGGTACCTGGTGCATGTGAAAGGAAAGAC	This study
*vfr*DR	GCCCAAGCTTGCGACCAGCCTGCACGAG	This study
PA4916PF	GCGAGCTCTCCTTGCTGCCCAGGCGCAGC	This study
PA4916PR	AACTGCAGTCACTTGCCGAAGGCGTGGCGGTGG	This study
PA4916F	AACTGCAGGGCGGTCTGAAGAGAGGAGTGACG	This study
PA4916R	CCCAAGCTTTGCCGCACCCGTTTGTCAGG	This study
*cyaA*Fown	GGGGTACCGCAGCGCATCCTCGCCAGCGGCGAG	This study
*cyaA*Ftac	GGGGTACCCTGGCCTTGCCTGTGCCCGGACGCCC	This study
*cyaA*R	CCCAAGCTTTCACTTGTCGTCATCGTCCTTGTAGTCTTGTTCCAGCAGCGCCTGGTTCAGCGCCG	This study
*cyaB*Fown	GGGGTACCTCGCCGAGTTCTACCCCTACTACCTGCAG	This study
*cyaB*Ftac	GGGGTACCATACACAGGGATCCTCTCCAGCGCATG	This study
*cyaB*R	CCCAAGCTTTCACTTGTCGTCATCGTCCTTGTAGTCGAGGATGACCTTGTCGCGCAGGCGTTCGG	This study
*nadD2*OF	GGAATTCCAAGCACTTGTACTACAAAATTTCGCAG	This study
*nadD2*ORHis	CCCAAGCTTTCAATGATGATGATGATGATG GCCCGCCTGGCGTTCGCCGCCATAGCAGTG	This study
*nadD2*ORFlag	CCCAAGCTTTCACTTGTCGTCATCGTCCTTGTAGTC GCCCGCCTGGCGTTCGCCGCCATAGCAGTG	This study
PA4918-20OF	GCTCTAGACAAGCGGAGGCTTCCATGAATCGCCCCAGC	This study
PA4918-20OR	CCCAAGCTTTCAGGGCGCCTTCGGCAGTTCGCGCTTGTG	This study
*cpdA*Fown	GGGGTACCCGCAGGCCTCGCGCCGGGTCGCGCTGAGCG	This study
*cpdA*R	CCCAAGCTTTCACTTGTCGTCATCGTCCTTGTAGTCGTATCCGGCGGTGTCGTAGTCCACTTC	This study
pET16b-*nadD2*F	TTTTCTCCATGGGCCGTGATGAAATAAGTTCCCGGATTCGCCGA	This study
pET16b-*nadD2*R	CCGCTCGAGGCCCGCCTGGCGTTCGCCGCCATAGCAG	This study
pET28a-*cyaB*_217-463_F	CATGCCATGGGCAAGAGCGTGCGCCTGGAAACCCAGC	This study
pET28a-*cyaB*_217-463_R	CCGCTCGAGGAGGATGACCTTGTCGCGCAGGCGTTCGG	This study
pMMB67EH-*cyaB*_217-463_F	GGGGTACCAAGAGCGTGCGCCTGGAAACCCAGC	This study
pMMB67EH-*cyaB*_217-463_R	CCCAAGCTTTCAATGATGATGATGATGATG GAGGATGACCTTGTCGCGCAGGCGTTCGG	This study
pMMB67EH-*cyaA*-hisR	CCCAAGCTTTCAATGATGATGATGATGATG TTGTTCCAGCAGCGCCTGGTTCAGCGCCG	This study
*cyaB*-SDF	AATTCATACACAGGGATCCTCTCCAGCGCATGGGTAC	This study
*cyaB*-SDR	CCATGCGCTGGAGAGGATCCCTGTGTATG	This study
*lac*P1F	GAATTCGCCCAATACGCAAACCGC	This study
*lac*P1R	GGATCCTCAGGCGAAAGGGGGATGTGCTG	This study
p_Tn7L_	ATTAGCTTACGACGCTACACCC	This study
p_glmS-up_	CTGTGCGACTGCTGGAGCTGA	This study
qPCR primer		
q*cyaA*F	CTTCAAGGAGCAGGTATTC	This study
q*cyaA*R	TTCGAGATGGCGATAGAC	This study
q*cyaB*F	GACCTGCTCAACAACTACC	This study
q*cyaB*R	GACGAACTTGTCGATGGT	This study
q*cpdA*F	GCGGATCGACCTGATTCTC	This study
q*cpdA*R	CTGCGGAAGCGTGTGTAG	This study
qPA4918F	GTCATCGAATACCTGAGG	This study
qPA4918R	GTTCTTCACGCAGTAGTC	This study
q*nadD2*F	GGTGTATTGCGAACCGATC	This study
q*nadD2*R	GCTTCCAGCAGGTCGATG	This study
q*exsA*F	CACGTCGGATAATCCTGATT	This study
q*exsA*R	TAGCGGAGAGGCATGAATA	This study
q*prpL*F	TATCGTATTTCGCCGACTCCC	This study
q*prpL*R	GCGAGTTGCCGTTGTTCAG	This study
q*toxA*F	CGAGATGGGCGACGAGTTG	This study
q*toxA*R	TGATGACCGTGGGCTTGATGT	This study
q*rpsL*F	CAAGCGCATGGTCGACAAGAG	This study
q*rpsL*R	ACCTTACGCAGTGCCGAGTTC	This study

*^a^F, forward; R, reverse; U, upstream of specific gene; D, downstream of specific gene; P, promoter of specific gene; own, native promoter of specific gene; tac, tac promoter; O, for over-expression plasmid construction; SD, Shine-Dalgarno sequence. ^b^The underlines are the sites of restriction enzymes.*

For the deletion of the *nrtR* gene, a 1,058 bp fragment immediately upstream of the *nrtR* start codon and a 1,308 bp fragment downstream of the *nrtR* stop codon were PCR amplified, digested with *Xba*I-*Eco*RI and *Hin*dIII-*Xba*I, respectively. The two fragments were then ligated into pEX18Tc that was digested with *Eco*RI and *Hin*dIII, resulting in pZF01. Similar manipulation was used to construct the *vfr*, *cyaA*, *cyaB*, *nadD2* and *nadD2*-*nrtR* deletion plasmids (detailed descriptions in Supplementary Text).

For the *nrtR* complementation plasmid, a 735 bp *nrtR*-containing fragment and a 465 bp fragment containing the promoter of *nadD2-nrtR* operon was amplified by PCR using PAK genomic DNA as template (primers shown in Table 2). The 735 bp and 465 bp PCR products were digested with *Pst*I/*Hin*dIII and *Pst*I/*Sac*I, respectively, and then ligated into the vector pUC18T-mini-Tn7T digested with *Hin*dIII and *Sac*I, resulting in pZF02. The plasmid was introduced into the Δ*nrtR* mutant by electroporation, along with the helper plasmid pTNS3 (Choi and Schweizer, 2006). Insertion of the *nrtR* gene into the chromosome was confirmed by PCR with primers P_Tn7L_ and P_glmS-up_ (primers shown in Table 2).

For overexpression of *nadD2*, a 643 bp *nadD2*-containing fragment with its putative Shine-Dalgarno (SD) sequence was PCR amplified using PAK genomic DNA as the template (primers shown in Table 2). The PCR product was digested with *Hin*dIII and *Eco*RI, and then ligated into a shuttle vector pMMB67EH which was digested with the same restriction enzymes, resulting in pMMB67-*nadD2*. pMMB67-*cyaA*, pMMB67-*cyaB*, pMMB67-4918-20 (encoding PA4918-4920), pMMB67EH-*cyaA*-His and pUCP24-*nadD2*-Flag were constructed with the similar manipulation. For the translational fusion of CyaB-Flag, *cyaB* with c-terminal Flag-tag and its upstream 500 bp region was PCR amplified using PAK genomic DNA as template (primers shown in Table 2), digested with *Hin*dIII and *Kpn*I, and then ligated into a promoterless pUCP20 (Li et al., 2016), resulting in pE1553b. Similar manipulation was used to construct the CyaA and CpdA translational fusion plasmids pE1553a and pE1553-*cpdA*. For expression of catalytic domain of CyaB, open reading frame from amino acids 217 to 463, which corresponds to the catalytic domain of CyaB (Fulcher et al., 2010), was PCR amplified and digested with *Kpn*I-*Hin*dIII. This digested fragment, together with the annealed native SD region of *cyaB* (primers shown in Table 2), was cloned into the pMMB67EH, resulting in pMMB67EH-*cyaB*_217-463_-His. The *lac*P1 reporter was created as described by Fulcher et al. (2010), except that the *lac*P1 promoter was cloned into the vector pDN19*lacZ*Ω.

### Western Blot Assay

Overnight bacterial cultures were diluted 50-fold into fresh LB with or without 5 mM EGTA. The *P. aeruginosa* strains were then cultured to an OD_600_ of 1.0 in a shaking incubator. Then the bacterial pellet and supernatant were separated by centrifugation at 13,000 ×*g* for 2 min. Supernatant and pellet samples from equivalent number of bacterial cells were mixed with SDS-PAGE loading buffer, boiled for 10 min at 99°C, separated on 12% SDS-PAGE (15% SDS-PAGE for ExsA-Flag), transferred onto a polyvinylidene difluoride (PVDF) membrane (Millipore), and probed with a rabbit polyclonal antibody against ExoS, the RNA polymerase beta subunit (RNAP, Abcam) or a mouse monoclonal antibody against Flag (Sigma). Signals were detected with the ECL-plus kit (Millipore).

### Cytotoxicity Assay

Bacterial cytotoxicity was determined by measuring detachment of mammalian cells after *P. aeruginosa* infection. 1.4 × 10^5^ HeLa cells (Deng et al., 2017) were seeded into each well of a 24-well plate and cultured in Dulbecco’s modified Eagle’s medium (DMEM) (Deng et al., 2017) containing 10% fetal bovine serum (FBS) (Deng et al., 2017), penicillin (100 μg/ml) and streptomycin (50 μg/ml) at 37°C with 5% CO_2_ the night before infection. Three hours before infection, cells were washed twice with phosphate-buffered saline (PBS) (Deng et al., 2017) and incubated in DMEM with 10% FBS. Log phase bacteria were used to infect HeLa cells at a multiplicity of infection (MOI) of 50. 50 mM cAMP was added into DMEM medium at the start of infection as indicated. Three hours post infection, the culture medium was removed from each well, and cells remaining attached were washed twice with PBS and stained with 500 μl 0.1% crystal violet in 10% ethanol for 15 min at 37°C. After discarding the staining solution, each well was washed twice with 1 ml distilled water and dried in air. A 200 μl volume of 95% ethanol was added into each well and incubated at room temperature for 30 min with gentle shaking. The dissolved crystal violet was subjected to measurement of absorbance at a wavelength of 590 nm.

### Murine Acute Pneumonia Model

Bacterial overnight culture was inoculated into fresh LB medium with 50-fold dilution and grown to an OD_600_ of 1.0. The bacteria were collected by centrifugation and adjusted to 1 × 10^9^ CFU/ml in PBS. The exact number of bacteria for each inoculum was further determined by serial dilution and plating. Six- to eight-week-old female BALB/c mice were anesthetized with 100 μl chloral hydrate (7.5%) by intraperitoneal injection, and then intranasally inoculated with 10 μl of bacterial suspension in each nostril, giving a total infection bacterial number of approximately 2 × 10^7^ per mouse. After 12 h, the mice were sacrificed and the lungs were dissected and homogenized in 1% protease peptone. The bacterial loads were determined by serial dilution and plating. The experimental results were analyzed with the GraphPad Prism software.

### Histology

Twelve hours post infection with *P. aeruginosa* strains or sterile PBS, mouse lungs were removed and fixed with 10% paraformaldehyde. Fixed tissues were dehydrated in grades of ethanol, embedded in paraffin, sectioned, and stained with hematoxylin and eosin. Images were taken with a 20× objective lens.

### Total RNA Isolation and RT-qPCR

Bacterial overnight culture was inoculated into fresh LB medium with 50-fold dilution and grown to an OD_600_ of 1.0 under T3SS inducing and non-inducing conditions. Total RNA was isolated using an RNA prep Pure cell/Bacteria Kit (Tiangen Biotech). cDNA was synthesized with a PrimeScript Reverse Transcriptase and random primers (Takara). The cDNA was mixed with indicated primers (shown in Table 2) and SYBR premix Ex Taq II (Takara). The 30S ribosomal protein encoding gene *rpsL* was used as an internal control.

### cAMP Assay

Intracellular cAMP concentration was measured as previously described (Fulcher et al., 2010). Overnight bacteria were subcultured with 50-fold dilution into LB and grown to an OD_600_ of 1.0. 1.5 ml of the bacteria were harvested by centrifugation at 13,000 ×*g* for 2 min at 4°C and washed twice with cold 0.9 M NaCl. Pellets were resuspended in 100 μl of 0.1 M HCl and incubated on ice for 10 min with occasional vortex to lyse bacteria. Cellular debris were removed by centrifugation at 13,000 ×*g* for 5 min at 4°C and the supernatant was used to measure intracellular cAMP using an enzyme-linked immunosorbant assay (ELISA kit, Cayman Chemical) following the manufacturer’s protocol for sample acetylation. For protein concentration determination, duplicate bacterial pellets were resuspended in 100 μl PBS and lysed by three freeze/thaw cycles followed by centrifugation at 13,000 ×*g* for 5 min at 4°C. The protein concentration of the supernatant was measured by the BCA protein assay (Beyotime Biotechnology). Assay values for cAMP levels were converted to intracellular concentrations per mg of protein.

### Co-immunoprecipitation Assay

The co-immunoprecipitation assay was performed as previously described with minor modifications (Shi et al., 2015). Δ*nadD2* containing pUCP24-*nadD2*-Flag and pMMB67EH-*cyaB*_217-463_-His, pMMB67EH-*cyaA*-His or the empty vector pMMB67EH were grown overnight and diluted 50-fold into fresh LB medium. When the OD_600_ reached 0.6, 1 mM IPTG was added to induce the expression of CyaB_217-463_-His or CyaA-His at 16°C for 18 h. Bacteria were harvested by centrifugation at 5,000 *g* for 10 min, resuspended in lysis buffer (20 mM Tris-HCl, 150 mM NaCl, 3 mM β-mercaptoethanol, 20 mM imidazole, 0.5% NP-40, pH 8.0) and lysed by sonication. Supernatants were collected by centrifugation and incubated with Ni-NTA agarose beads for 1 h at 4°C. The beads were washed five times with the lysis buffer and boiled in the SDS-PAGE loading buffer. Samples were separated by 15% SDS-PAGE (CyaB_217–463_-His) or 12% SDS-PAGE (CyaA-His) and probed with an anti-Flag (Sigma) or anti-His antibody (Millipore).

### Expression and Purification of CyaB_217-463_ or NadD2 Protein

The full-length *nadD2* or catalytic domain of the *cyaB* (amino acid positions 217–463) was PCR amplified from PAK chromosomal DNA with primers shown in Table 2 and cloned into pET16b or pET28a, resulting in pET16b-*nadD2* or pET28a-*cyaB*_217-463_, respectively. Overnight culture of the *E. coli* strain BL21 (DE3) carrying pET16b-*nadD2* or pET28a-*cyaB*_217-463_ was subcultured with 50-fold dilution into 500 ml fresh LB medium at 37°C. When the OD_600_ reached 0.6, 1 mM IPTG was added to induce the protein expression at 16°C for 16 h. The bacteria were collected by centrifugation at 4°C, 5,000 ×*g*, for 20 min and resuspended in the lysis buffer (20 mM Tris-HCl, 150 mM NaCl, 3 mM β-mercaptoethanol, 10 mM imidazole, 0.5% NP-40, pH 8.0), followed by sonication. The bacterial lysate was centrifuged at 15,000 rpm for 20 min at 4°C and the supernatant was applied to a Ni-NTA column (Qiagen). After the Ni-NTA column was washed four times with the lysis buffer containing 20 mM imidazole, the protein was eluted with 400 mM imidazole prepared in lysis buffer, followed by dialysis against enzymatic reaction buffer (100 mM NaCl, 20 mM Tris-HCl, 10 mM MgCl_2_). The purified protein was examined by SDS-PAGE, and quantified by BCA protein assay (Beyotime Biotechnology). Expression and purification of the LasR protein was described in a previous report (Fan et al., 2018).

### Adenylate Cyclase Activity Assays

The adenylyl cyclase activity assays were performed as previously described with minor modifications (Topal et al., 2012). Briefly, the reaction was performed in a 50 μl volume of 100 mM NaCl, 20 mM Tris-HCl (pH 7.0), 10 mM MgCl_2_, 5 mM ATP, and 2.0–2.25 μg purified CyaB protein, with or without 4.5–5.5 μg NadD2 or LasR protein. Samples were incubated at 30°C for 30 min, and then heated at 95°C for 4 min. The reaction mixtures were centrifuged at 15,000 rpm for 1 min, followed by filtration with a 0.22 μm filter, and then the cAMP concentrations were determined using an ELISA kit (Cayman Chemical) according to the manufacturers’ instructions.

### Other Methods

The *nrtR* gene knock out was generated by homologous recombination as described previously (Hoang et al., 1998). β-galactosidase activity assay was conducted to determine the *lac*P1 promoter transcriptional activity as described before (Wu et al., 2004). The measurement of ATP concentration was carried out following the manufacturer’s instruction (Beyotime Biotechnology). DNA manipulations were performed according to Molecular Cloning (Sambrook et al., 1989).

### Statistical Analysis

GraphPad Prism software was used to perform the statistical analyses. Results were analyzed by Mann–Whitney test or the Student’s *t*-test (two-tailed).

## Results

### The PA4916 Mutant Is Defective in T3SS-Dependent Cytotoxicity

**FIGURE 1:**
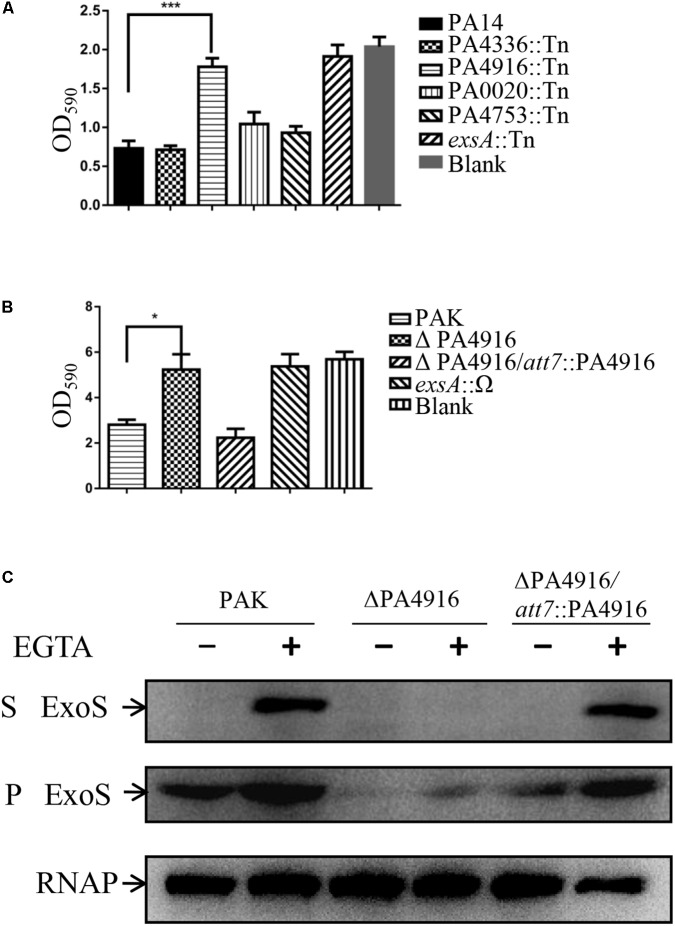
Cytotoxicity of indicated strains and the role of PA4916 in the expression and secretion of ExoS. **(A,B)** HeLa cells were infected with indicated strains at a MOI of 50. Three hours post infection, cells attached to the 24-well plate were washed with PBS and stained with crystal violet. The cell associated crystal violet was dissolved in ethanol and quantified by measuring OD_590_. HeLa cells with no bacterial infection served as a control. **(C)** Bacteria were cultured to an OD_600_ of 1.0 in LB with or without 5 mM EGTA. Proteins in supernatants and pellets from equivalent bacterial cells were loaded onto SDS-PAGE gels and probed with an antibody against ExoS or RNA polymerase beta subunit. S, supernatant; P, pellet.

In our previous screen for T3SS related genes, we found 23 genes that affect T3SS (Li et al., 2013). Among them, PA0020, PA3202, PA4336, PA4630, PA4916, and PA4753 encoded products are annotated as hypothetical proteins^1^ (Winsor et al., 2016) with unknown biological functions. To confirm their relationships with T3SS as well as exclude strain specific phenotype, mutants with Transposon (Tn) insertions in the PA0020, PA4336, PA4916, and PA4753 from the PA14 Non-Redundant Transposon Insertion Mutant Set (PA14NR Set) were selected for further tests (Liberati et al., 2006), however, mutant of the PA3202 or PA4630 is not available in the PA14NR Set. As T3SS plays a major role in cytotoxicity (Hauser et al., 1998), we infected HeLa cells with those mutants. Detached cells due to cytotoxicity were washed away, and the remaining cells were observed and quantified by crystal violet staining. Similar to wild type PA14, mutant strains of PA0020, PA4336 and PA4753 detached most of the HeLa cell within 3 h, whereas the ΔPA4916 mutant caused minimal detachment (Figure 1A), indicating a defective cytotoxicity.

To further confirm the role of PA4916, the whole open reading frame of PA4916 was deleted from the PAK chromosome via DNA recombination, resulting in ΔPA4916. As shown in Figure 1B, this mutant also displayed a reduced cytotoxicity, which was restored nearly to that of wild type by complementation with an intact PA4916 gene.

### PA4916 Is Required for ExoS Expression and Involved in Pathogenesis of *P. aeruginosa*

To verify whether the reduced cytotoxicity is due to a defective T3SS, wild type PAK and the ΔPA4916 mutant were grown under T3SS inducing and non-inducing conditions (in the presence and absence of 5 mM EGTA), and the expression and secretion of ExoS were examined by Western blot. Under T3SS inducing condition, the expression and secretion of ExoS were highly induced in the wild type PAK, however, faint ExoS was observed in the pellet and not detected in the supernatant of ΔPA4916. Complementation with an intact PA4916 gene restored the expression and secretion of ExoS in the ΔPA4916 mutant background (Figure 1C).

**FIGURE 2:**
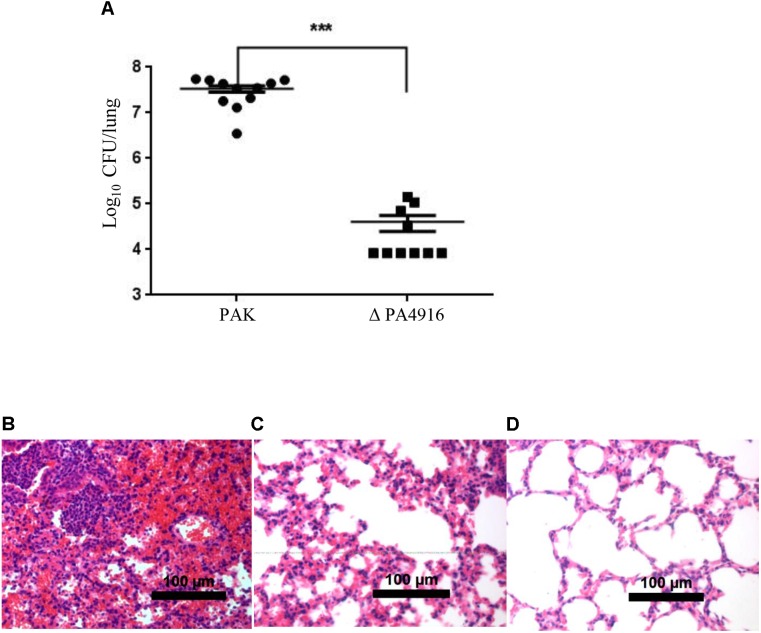
Role of PA4916 in the mouse acute pneumonia model. **(A)** Bacteria were grown to an OD_600_ of 1.0. Female BALB/c mice (6- to 8-week-old) were inoculated intranasally with 2 × 10^7^ CFU of wild-type PAK or its isogenic ΔPA4916 mutant. After 12 h, the mice were sacrificed, and the lungs were dissected and homogenized. The bacteria load in each lung was determined by serial dilution and plating. ^∗∗∗^*P* < 0.001, by Mann–Whitney test. **(B–D)** Pathology sections of lungs infected with indicated strains. Mice were infected with 2 × 10^7^ CFU of wild type PAK **(B)**, ΔPA4916 mutant **(C)** or sterile PBS **(D)**. Lungs from infected mice were removed, fixed, sectioned, and stained with hematoxylin and eosin. Images were taken with a 20× objective lens.

The T3SS plays an important role in acute infections (Sadikot et al., 2005). The functional connection between PA4916 and the T3SS promoted us to examine its role in the pathogenesis of a mouse acute pneumonia model. Six- to eight-week-old female BALB/c mice were infected intranasally with 2 × 10^7^ CFU of wild type PAK or the ΔPA4916 mutant. Twelve hours post infection, lungs were isolated and homogenized. Bacterial loads were determined by serial dilution and plating. Compared to the wild type PAK strain, the number of ΔPA4916 mutant isolated from the lungs was significantly lower (Figure 2A), suggesting a defective virulence. Reduced growth rate in the ΔPA4916 mutant might lead to a reduced virulence; however, the deletion mutant ΔPA4916 showed a rate of growth indistinguishable from that of wild type PAK strain when cultured in LB medium (Supplementary Figure S1).

To further validate the role of PA4916 in the bacterial virulence, the lungs infected by wild type or ΔPA4916 mutant were observed following histological section and staining. Lungs from mice infected with PAK for 12 h had significant neutrophil infiltration, edema and tissue damage (Figure 2B). Most of the airways in the lungs of these mice were completely occluded with neutrophil and pyocyte infiltration. In contrast, infections with the ΔPA4916 mutant showed significantly reduced inflammatory characteristics (Figure 2C), with substantially fewer neutrophils present in the alveolar spaces, compared to the infections with PAK, though the inflammation was more intense compared to the PBS instilled control (Figure 2D). The extent of inflammation caused by the ΔPA4916 mutant correlated with the ability of this strain to colonize the lungs of infected mice. A recent study has also shown that PA4916 plasposon mutagenesis abrogated virulence of a robust mucoid *P. aeruginosa* cystic fibrosis airway isolate and named PA4916 as NrtR (Okon et al., 2017). So we further explored the regulation mechanism of PA4916 on the T3SS and referred PA4916 as NrtR hereafter.

### Plasmid-Expressed *exsA* Restores the T3SS in Δ*nrtR* Mutant

**FIGURE 3:**
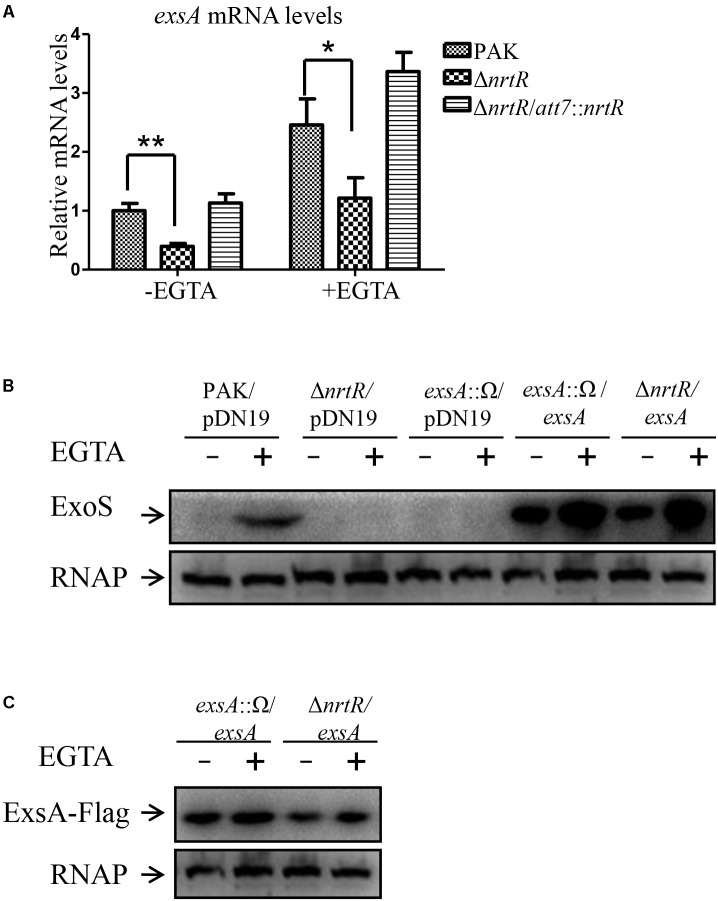
Plasmid mediated expression of *exsA* restored T3SS in the Δ*nrtR* mutant. **(A)** The relative *exsA* mRNA levels in PAK, the Δ*nrtR* mutant and Δ*nrtR*/*att7*:: *nrtR* strain. Total RNA was isolated under T3SS inducing and non-inducing conditions and the *exsA* mRNA levels were determined by real-time PCR using *rpsL* as the internal control. ^∗^*P* < 0.05, ^∗∗^*P* < 0.01, by Student’s *t*-test. **(B)** Bacteria harboring an *exsA*-Flag driven by a *lac* promoter or the empty vector pDN19 were grown to an OD_600_ of 1.0 in LB with or without EGTA. Proteins from equivalent number of bacterial cells of indicated strains were separated on SDS-PAGE and probed with an anti-ExoS antibody or an anti-RNA polymerase beta subunit antibody. Expression levels of the ExsA-Flag were determined with an anti-FLAG antibody **(C)**.

ExsA is a central regulator of T3SS (Hovey and Frank, 1995). To investigate whether NrtR regulates T3SS through ExsA, total RNA was isolated and the mRNA levels of *exsA* were compared between PAK and the Δ*nrtR* mutant. As shown in Figure 3A, under both T3SS inducing and non-inducing conditions, the *exsA* mRNA level was significantly decreased in the Δ*nrtR* mutant, which was restored by complementation with a *nrtR* gene. To further confirm the role of *exsA* in the NrtR mediated regulation of T3SS, a plasmid carrying an *exsA* gene driven by a *lac* promoter (Totten and Lory, 1990; Li et al., 2013) was introduced into the Δ*nrtR* or an *exsA*::Ω mutant. Plasmid-expressed ExsA restored the expression of ExoS in both the *exsA*::Ω and Δ*nrtR* mutants (Figure 3B). Although the expression of ExoS was similar in the *exsA*::Ω/*exsA* and Δ*nrtR/exsA* mutants, the ExsA amount in the Δ*nrtR/exsA* mutant was much less than that in the *exsA*::Ω/*exsA* mutant (Figure 3C). Considering the fact that *lac* promoter is controlled by catabolite repression and T3SS is regulated by the cAMP-Vfr signaling pathway, the decreased ExsA amount may indicate a reduced cAMP level in the Δ*nrtR* mutant strain.

### The cAMP-Vfr Signaling Pathway Is Involved in NrtR Mediated Regulation of T3SS

**FIGURE 4:**
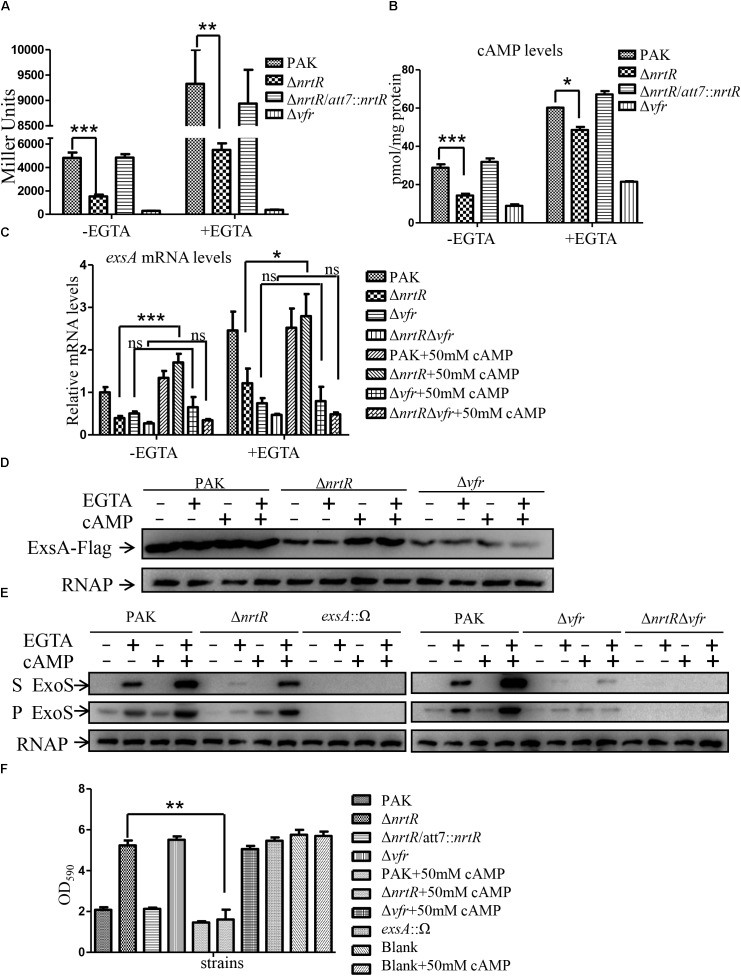
Decreased cAMP contributed to the T3SS defect in the Δ*nrtR* mutant. **(A,B)** cAMP level was decreased in Δ*nrtR* mutant and Δ*vfr* mutant. β-galactosidase assay was used to examine the transcriptional activity of *lac*P1 promoter fused to a *lacZ* gene in indicated strains under T3SS inducing and non-inducing conditions **(A)**. **(B)** Intracellular cAMP levels were measured using an ELISA kit. Error bars represent standard deviations. ^∗^*P* < 0.05, ^∗∗^*P* < 0.01, ^∗∗∗^*P* < 0.001 by Student’s *t-*test. **(C–F)** Exogenous addition of cAMP recovers the expression of ExsA, ExoS and cytotoxicity of the Δ*nrtR* mutant, while not of or Δ*nrtR*Δ*vfr* mutant. **(C)** Relative mRNA levels of *exsA* in indicated strains with or without cAMP addition at the beginning of subculture in the presence or absence of 5 mM EGTA, with *rpsL* as an internal control. ns, not significant, ^∗^*P* < 0.05, ^∗∗∗^*P* < 0.001 by Student’s *t-*test. **(D)** Indicated strains containing an *exsA*-Flag driven by its native promoter were grown at 37°C with or without 5 mM EGTA and 50 mM cAMP as indicated. Protein samples from equal number of bacteria were separated by SDS-PAGE and probed with an anti-Flag antibody or an anti-RNA polymerase beta subunit antibody. **(E)** Expression of ExoS in indicated strains were grown with or without 5 mM EGTA and 50 mM cAMP. The protein levels were detected with an antibody against ExoS or RNA polymerase beta subunit. S, supernatant; P, pellet. **(F)** Cytotoxicity of indicated strains in the presence or absence of 50 mM cAMP. HeLa cells were infected with indicated strains at a MOI of 50. 50 mM final concentration of cAMP was added to DMEM medium as indicated. Three hours post infection, cells attached to the 24-well plate were washed with PBS and stained with crystal violet. The cell associated crystal violet was dissolved in ethanol and quantified by measuring OD_590_. HeLa cells with no bacterial infection (blank and blank+50 mM cAMP) served as a control. ^∗∗^*P* < 0.01, by Student’s *t-*test.

To investigate if the cAMP-Vfr signaling pathway is involved in the NrtR mediated T3SS regulation, we initially compared the cAMP contents between PAK and Δ*nrtR* mutant with a transcriptional fusion of the *lac*P1 promoter and a *lacZ* gene (*lac*P1-*lacZ*), whose expression has been shown to correlate to intracellular cAMP levels (Fulcher et al., 2010). A Δ*vfr* mutant was included as a control of Δ*nrtR* mutant (Fulcher et al., 2010). As expected, the LacZ levels in the Δ*nrtR* mutant under both T3SS inducing and non-inducing conditions were lower than those of the PAK strain, which were restored by complementation with a *nrtR* gene (Figure 4A). This result was further confirmed by direct measurement of intracellular cAMP levels with a cAMP ELISA detection kit (Figure 4B). A previous study has shown that exogenous addition of 50 mM cAMP restored the phenotypes of an adenylate cyclase mutant of *P. aeruginosa* (Fulcher et al., 2010). Therefore, we constructed a c-terminus Flag-tagged ExsA driven by its native promoter and examined the effect of exogenous addition of 50 mM cAMP on the T3SS in the Δ*nrtR* mutant. As shown in Figure 4C,D, exogenous addition of cAMP restored the *exsA* expression levels in the Δ*nrtR* mutant at both transcriptional and protein levels, but not in the Δ*vfr* mutant. Furthermore, the expression and secretion of ExoS and cytotoxicity of Δ*nrtR* were restored to wild type level by exogenous addition of 50 mM cAMP (Figure 4E,F). As expected, exogenous addition of 50 mM cAMP did not affect the expression and secretion of ExoS and cytotoxicity of the Δ*vfr* mutant (Figure 4E,F). In addition, Δ*nrtR*Δ*vfr* double mutant strain was constructed. The *exsA* transcriptional level, as well as the expression and secretion of ExoS of Δ*nrtR*Δ*vfr* were compared with Δ*nrtR* and Δ*vfr* mutant. The results showed that, like Δ*vfr* mutant, exogenous cAMP addition did not affect *exsA* transcriptional level, as well as the expression and secretion of T3SS in Δ*nrtR*Δ*vfr* double mutant strain (Figure 4C,E). These results demonstrate that NrtR regulates T3SS through the cAMP-Vfr signaling pathway in *P. aeruginosa*, likely by altering the intracellular cAMP level. Furthermore, twitching motility and the expression levels of *toxA* and *prpL* which were demonstrated to be affected by cAMP were examined in the Δ*nrtR* mutant (Wolfgang et al., 2003). As expected, the expression levels of both *toxA* and *prpL* were decreased significantly in Δ*nrtR* mutant (Supplementary Figure S2). However, the twitching motility of the Δ*nrtR* mutant showed no detectable change compared to the wild type PAK strain (Supplementary Figure S2).

### Decreased Intracellular cAMP Is Not Caused by Altered Expression of CyaA or CyaB in the Δ*nrtR* Mutant

**FIGURE 5:**
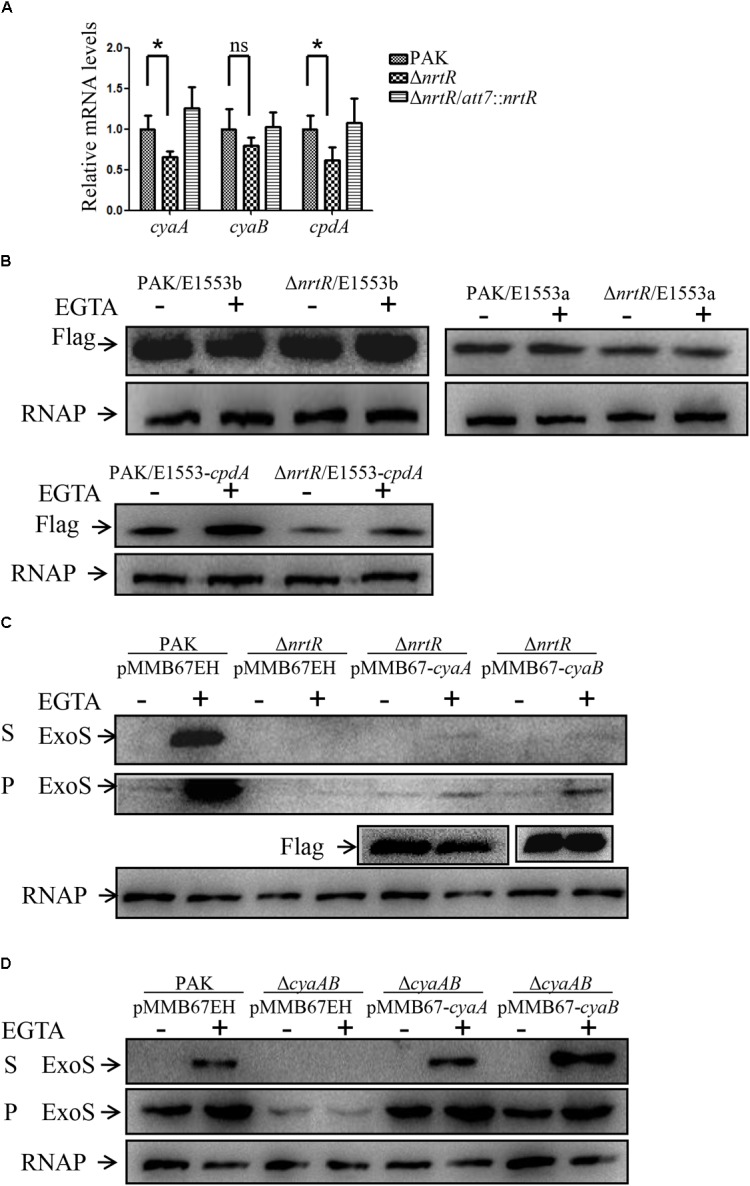
The decrease of cAMP is not caused by changed expression of CyaA, CyaB or CpdA. **(A)** Relative mRNA levels of *cyaA*, *cyaB*, and *cpdA*. Total RNA of indicated strains was isolated and the mRNA levels of these genes were determined by real time PCR with *rpsL* serving as an internal control. ns, not significant, ^∗^*p* < 0.05 by Student’s *t*-test. **(B)** Indicated strains containing a *cyaA*-Flag (*cyaB*-Flag or *cpdA*-Flag) driven by its native promoter were grown at 37°C with or without 5 mM EGTA until OD_600_ of 1.0. Samples from equal number of bacteria were separated by SDS-PAGE and probed with an anti-Flag antibody or an anti-RNA polymerase beta subunit antibody. **(C,D)** Indicated strains containing a *cyaA*-Flag or *cyaB*-Flag driven by a *tac* promoter or the empty vector pMMB67EH were grown to an OD_600_ of 1.0 in LB with 1 mM IPTG with or without 5 mM EGTA. Proteins from equivalent bacterial cells of indicated strains were separated by SDS-PAGE and probed with an anti-ExoS antibody, an anti-Flag antibody or an anti-RNA polymerase beta subunit antibody. S, supernatant; P, pellet.

In *P. aeruginosa*, cAMP is synthesized by the adenylate cyclases CyaA and CyaB (Wolfgang et al., 2003; Marsden et al., 2016). To explore if the reduced cAMP level is caused by decreased expression of the adenylate cyclases in Δ*nrtR* mutant, we determined the expression levels of the two genes by RT-qPCR. As shown in Figure 5A, the mRNA level of *cyaA* was lower in the Δ*nrtR* mutant than those in both wild type PAK and the complemented strain, while the *cyaB* was similar among these three strains. To confirm this observation, C-terminal Flag-tagged CyaA or CyaB driven by their respective native promoters, were transformed into PAK and the Δ*nrtR* mutant, and their protein expression levels were examined by Western blot assay. Consistent with the RT-qPCR result, similar level of CyaB-Flag, while slightly lower level of CyaA-Flag protein were observed in the Δ*nrtR* mutant compared to that in PAK (Figure 5B). To further understand if the observed slight reduction of the adenylate cyclase is the cause of the decreased cAMP in Δ*nrtR* mutant, the functional C-terminal Flag tagged CyaA or CyaB was driven by a *tac* promoter and transformed into the Δ*nrtR* mutant. Similar levels of expression and secretion of ExoS were observed by Western blot assay between Δ*nrtR*/pMMB67EH and Δ*nrtR*/pMMB67-*cyaA* or Δ*nrtR*/pMMB67-*cyaB* (Figure 5C,D). These results suggest that the reduced cAMP level in the Δ*nrtR* strain is not due to alteration of adenylate cyclases expression.

### Decreased Intracellular cAMP Is Not Caused by Altered Expression of CpdA in the Δ*nrtR* Mutant

Since we did not get evidence for an effect of altered expression of adenylate cyclases on the T3SS in the Δ*nrtR* mutant, we next wanted to investigate if the degradation of cAMP was affected in the Δ*nrtR* mutant. To date, CpdA is the only known phosphodiesterase degrading cAMP in *P. aeruginosa* (Fuchs et al., 2010). One possibility for the observed decrease in cAMP levels and subsequent T3SS in the Δ*nrtR* mutant could be that CpdA expression is upregulated in the Δ*nrtR* mutant. This would in turn result in an increased degradation of cAMP and a decrease in T3SS. To test this possibility, total RNA was isolated and mRNA levels of *cpdA* were compared among wild type PAK, Δ*nrtR* mutant and Δ*nrtR* complemented strain by RT-qPCR. As shown in Figure 5A, the mRNA level of *cpdA* was lower, rather than higher, in the Δ*nrtR* mutant than those in both wild type PAK and complemented strain. To confirm this observation, C-terminal Flag-tagged CpdA driven by its native promoter, was transformed into PAK and the Δ*nrtR* mutant, and their protein expression levels were examined by Western blot assay. Consistent with the RT-qPCR result, lower CpdA-Flag protein level were observed in the Δ*nrtR* mutant than that in PAK (Figure 5B). These results thus suggest that decreased cAMP and subsequent T3SS does not occur through increased CpdA-mediated cAMP degradation.

### Increased NadD2 Level Contributes to the Decreased cAMP and T3SS in the Δ*nrtR* Mutant

**FIGURE 6:**
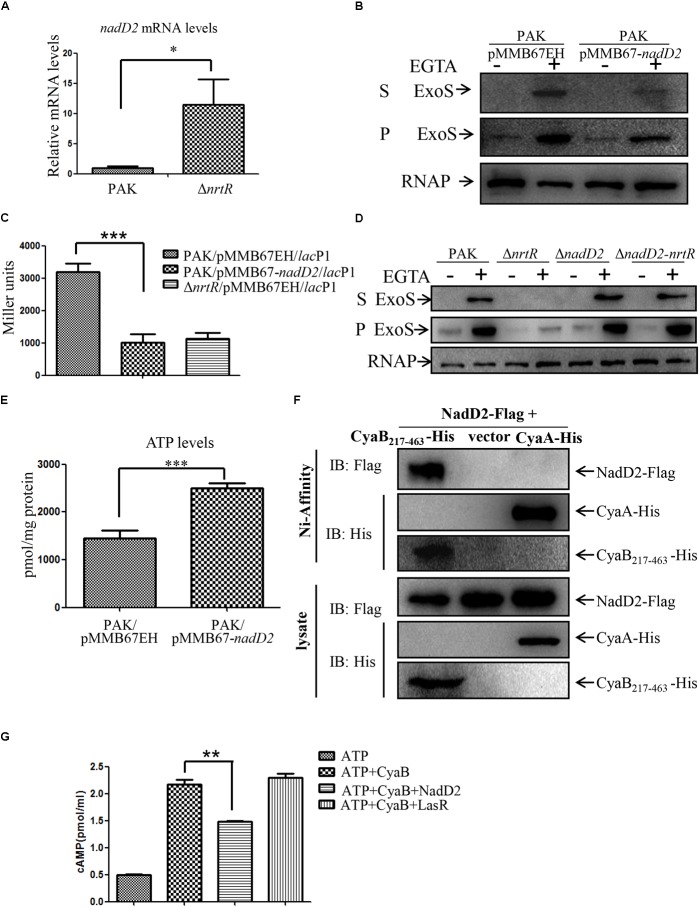
Increased expression of NadD2 contributes to the decreased intracellular cAMP and expression of T3SS in the Δ*nrtR* mutant. **(A)** Relative mRNA levels of *nadD2*. Total RNA of indicated strains was isolated and mRNA levels of *nadD2* were determined by real time PCR with *rpsL* serving as an internal control. Data represents the mean ± standard deviation. ^∗^*p* < 0.05 by Student’s *t*-test. **(B)** PAK containing *nadD2* driven by a *tac* promoter or the empty vector pMMB67EH were grown to an OD_600_ of 1.0 in LB containing 1 mM IPTG with or without 5 mM EGTA. Proteins from equivalent bacterial cells of indicated strains were separated by SDS-PAGE and probed with an antibody against ExoS or RNA polymerase beta subunit. S, supernatant; P, pellet. **(C)** The cAMP levels were decreased in the PAK/pMMB67-*nadD2* strain. β-Galactosidase assay was used to examine the LacZ level driven by the *lac*P1 promoter in indicated strains with 1 mM IPTG induction. Error bars represent standard deviations. ^∗∗∗^*P* < 0.001 by Student’s *t-*test. **(D)** Indicated bacteria were cultured to an OD_600_ of 1.0 in LB with or without 5 mM EGTA. Proteins in supernatants and pellets from equivalent bacterial cells were loaded onto SDS-PAGE gels and probed with an antibody against ExoS or RNA polymerase beta subunit. S, supernatant; P, pellet. **(E)** ATP levels in indicated strains. ^∗∗∗^*P* < 0.001 by Student’s *t-*test. **(F)** Interaction between NadD2 and CyaB_217-463_ or CyaA. Δ*nadD2* carrying pUCP24-*nadD2*-Flag with pMMB67EH-*cyaB*_217-463_-His, pMMB67EH-*cyaA*-His or pMMB67EH were grown to an OD_600_ of 0.6 and incubated with 1 mM IPTG for 18 h at 16°C. Bacteria were lysed and subjected to chromatography with Ni-NTA beads. His-tagged CyaB_217–463_ or CyaA and FLAG-tagged NadD2 were detected by Western blot assay. **(G)** Inhibition of CyaB adenylyl cyclase activity by NadD2. 2.0–2.25 μg CyaB was incubated with 5 mM ATP. 4.5–5.5 μg NadD2 or LasR was added as indicated. After 30 min at 30°C, the cAMP levels were measured using an ELISA kit. Error bars represent standard deviations. ^∗∗^*P* < 0.01 by Student’s *t-*test.

*NrtR* encodes a transcriptional regulator which binds to the DNA intergenic region between the *nadD2*- *nrtR* and PA4918-4920 operons to repress their expression (Okon et al., 2017). RT-qPCR assay showed that the mRNA levels of *nadD2* and PA4918 in the Δ*nrtR* mutant were much higher than those in PAK (Figure 6A and Supplementary Figure S3A). Thus, we examined whether NadD2 or PA4918-4920 operon was involved in the NrtR mediated regulation of T3SS. Overexpression of *nadD2* in wild type PAK reduced the expression and secretion of the ExoS, as well as the intracellular cAMP level (Figure 6B,C). However, overexpression of PA4918-4920 operon in PAK showed no inhibitory effect on the expression and secretion of T3SS (Supplementary Figure S3B). In addition, deletion of *nadD2* in wild type PAK did not affect the expression and secretion of ExoS, whereas deletion of *nadD2* restored the expression and secretion of ExoS in the Δ*nrtR* mutant (Figure 6D). These results indicate that the increased NadD2 level might be responsible for the decreased cAMP and T3SS in the Δ*nrtR* mutant. NadD2 is an ATP consuming enzyme (Okon et al., 2017). Adenylate cyclases catalyzed the synthesis of cAMP from ATP. Reduced ATP availability due to increased NadD2 level might result in the decreased cAMP level in PAK/pMMB67-*nadD2*. However, the PAK/pMMB67-*nadD2* strain displayed a higher ATP level than the strain with an empty vector (PAK/pMMB67EH) (Figure 6E).

Since the decreased cAMP level is not caused by the changed expression of the adenylate cyclases in the Δ*nrtR* mutant, it is possible that their enzymatic activities might be affected by NadD2. To test this possibility, a CyaA-His or a CyaB_217-463_-His fusion protein (the catalytic domain of CyaB without the transmembrane region), was constructed and overexpressed in Δ*nadD2* carrying a NadD2-Flag fusion protein. The His-Tagged CyaA and CyaB_217-463_ were purified with Ni-affinity chromatography. As shown in Figure 6F, NadD2-Flag was co-purified with the CyaB_217-463_-His, but not with CyaA-His.

Next, we examined whether NadD2 directly represses the adenylyl cyclase activity of CyaB. CyaB_217-463_ and NadD2 were expressed in *E. coli* and purified (Supplementary Figure S4A). The purified NadD2 was not contaminated by ATPase or phosphodiesterase (Supplementary Figures S4B,C). The purified catalytic domain of CyaB (CyaB_217-463_) was incubated with ATP with or without NadD2 and the cAMP level was determined with a cAMP ELISA kit (Cayman Chemical). An unrelated protein LasR was used as a negative control. As shown in Figure 6G, the cAMP level was reduced by the presence of NadD2 but not LasR, indicating a repression of the adenylyl cyclase activity by the NadD2. These results suggest that NadD2 might suppress the enzymatic activity of CyaB.

## Discussion

In the present study, we identified that NrtR is required for the T3SS and involved in pathogenesis of *P. aeruginosa* in a murine acute pneumonia model. Further experimental results demonstrated that NrtR regulates expression of T3SS through the cAMP/Vfr signaling system. *NadD2*, which is in the same operon of *nrtR* and repressed by NrtR, is involved in the NrtR mediated regulation of the T3SS by inhibition of adenylyl cyclase activity of CyaB in *P. aeruginos*a.

In our previous study, we identified PA0020, PA4336, PA4916 and PA4753 as T3SS related genes by screening Tn insertion mutant library of PAK with an ELISA assay (Li et al., 2013). While in this study, only the *nrtR* mutant in PA14 background displayed a significant change in T3SS related cytotoxicity. The previous screening by ELISA was performed with EGTA as the T3SS inducing condition, whereas in the cytotoxicity assay, contact with host cell is the inducing signal. The different results in the two tests indicate that PA0020, PA4336 and PA4753 might play different roles in bacterial response to the two signals. In addition, the differences of the Tn insertion sites in each of the genes might cause different effects on the gene function, thus leading to different phenotypes. Further studies are required to fully understand the functions of those genes.

*NrtR* encodes a putative ADP-ribose pyrophosphatase with a Nudix hydrolase domain. Nudix protein specifically hydrolyzes varieties of substrates with a common structure of a Nucleoside diphosphates linked to moiety, X, such as (d) NTPs, coenzymes and capped RNAs (O’Handley et al., 1996; Cartwright et al., 2000; Song et al., 2013). It has been reported that some of these proteins may play important regulatory roles in response to stress, invasion to host cell and in pathogenesis (Edelstein et al., 2005; Urick et al., 2005; Liu et al., 2012). A recent study reported that *nrtR* of *P. aeruginosa* PA14 encodes a transcriptional repressor, which has no ADP-ribose pyrophosphatase activity and can bind to the DNA intergenic region between *nadD2-nrtR* and PA4918-4920 operons to repress their expression (Okon et al., 2017). Consistent with this, our study demonstrated that the transcriptional levels of *nadD2* and PA4918 increased 11- and 46-fold, respectively, in the Δ*nrtR* mutant (Figure 6A and Supplementary Figure S3A). Overexpression of NadD2 repressed the expression of T3SS in PAK strain, whereas overexpression of PA4918-4920 in PAK did not show any inhibitory effect on the expression of T3SS (Supplementary Figure S3B), thus NrtR positively controls the expression of T3SS in *P. aeruginosa* through repression of *nadD2* specifically. *nadD2* deletion restored the expression and secretion of T3SS in Δ*nrtR* mutant, but had no influence on the expression and secretion of T3SS in wild type PAK strain, indicating that the altered expression level of NadD2 is not sufficient to repress the expression of T3SS in wild type PAK strain.

*NadD2*, encoding a nicotinate mononucleotide adenylyltransferase, is located downstream of the transcriptional repressor NrtR and upstream of the PA4918-4920 (*pncA*-*pncB1*-*nadE*) operon. The interaction between NadD2 and other proteins were predicted using the STRING database^2^), a pre-computed database to predict both physical and functional interactions. The high confidence score (>0.7) exist between NadD2 and proteins encoded by its neighboring genes on the chromosome. It has been demonstrated that *pncA*, *pncB1*, and *nadE* encode the nicotinamidase, nicotinate phosporibosyltransferase and Nad synthase, respectively (Okon et al., 2017). Therefore, NrtR negatively regulates the salvage pathway I of the NAD biosynthesis. In addition, the co-immunoprecipitation assay in the present study suggests that NadD2 interacts with CyaB, but not with CyaA, indicating that NadD2 influences the cAMP production through inhibition of the adenylyl cyclase activity of CyaB. However, the inability of functional CyaA to complement the expression of T3SS in Δ*nrtR* mutant suggests that NrtR may also influence the adenylyl cyclase activity of CyaA indirectly.

cAMP, as an important second messenger, has been shown to regulate the T3SS, exotoxin A, protease IV and type IV pili biosynthesis (Wolfgang et al., 2003). As the cAMP was decreased in the Δ*nrtR* mutant, we also tested the twitching motility and the expression levels of *toxA* and *prpL* in the Δ*nrtR* mutant. As expected, the expression levels of both *toxA* and *prpL* were decreased significantly in Δ*nrtR* mutant. However, the twitching motility of the Δ*nrtR* mutant showed no detectable change compared to the wild type PAK strain (Supplementary Figure S2). It might be possible that the T3SS is more sensitive to changes in the cAMP level than twitching motility. The generation of cAMP in *P. aeruginosa* relies on CyaA and CyaB, while its degradation relies on phosphodiesterase CpdA (Fuchs et al., 2010). Expression of CpdA is lower in the Δ*nrtR* mutant, which may be caused by decreased levels of intracellular cAMP, as the *cpdA* can be directly activated by Vfr in response to intracellular cAMP as a feedback loop (Fuchs et al., 2010).

The intracellular cAMP levels are modulated by calcium concentration (Inclan et al., 2011), and the EGTA-induced calcium depletion has been shown to increase intracellular cAMP levels in *P. aeruginosa* (Wolfgang et al., 2003). Consistent with these results, our study demonstrated that intracellular cAMP levels were increased in both wild type PAK and Δ*nrtR* mutant under EGTA inducing condition, even in the Δ*vfr* mutant. Considering that EGTA addition did not affect the expression of adenylate cyclases in both PAK and Δ*nrtR* mutant, this increase suggests that EGTA might increase the activity of adenylate cyclases. The fact that the activation of *lac*P1 was Vfr-dependent in *P. aeruginosa* might result in no obvious increase of reporter activity in Δ*vfr* mutant under EGTA inducing condition (Fulcher et al., 2010). In contrast to the almost eliminated reporter activity, the Δ*vfr* mutant displayed an approximately 50% reduction in intracellular cAMP compared to the PAK strain, which is consistent with previous report (Fulcher et al., 2010).

The T3SS of *P. aeruginosa* can be induced by EGTA addition (calcium depletion) (Wolfgang et al., 2003). Previous studies reported that most secretion apparatus component and effector genes were regulated by calcium depletion except for the ExsA, whose expression was relatively unaffected (Wolfgang et al., 2003). Inconsistent with this finding, in our study, the mRNA levels of *exsA* showed a significant increase under EGTA inducing condition. This may be caused by the different experimental methods used in these two studies (transcriptomic analysis vs. RT-qPCR). Furthermore, the ETGA-dependent increase of ExsA protein levels was demonstrated previously (Intile et al., 2014; Ince et al., 2015). However, in contrast to the previous reports and our transcriptional levels of *exsA*, in this study, the protein levels of ExsA were not affected by EGTA addition both in PAK and the Δ*nrtR* mutant. In the previous studies, *P. aeruginosa* was grown in Trypticase soy broth, while we cultured bacteria in L-broth medium. The difference between the transcriptional levels and protein levels of *exsA* might be due to the sensitivity of RT-qPCR or some unknown post-transcriptional regulatory mechanism.

Recently, Okon et al. (2017) demonstrated that NrtR regulates nicotinamide adenine dinucleotide (NAD) biosynthesis and is involved in the virulence of a *P. aeruginosa* clinical isolate. Our study revealed novel functions of NrtR and NadD2 in the cAMP biosynthesis in *P. aeruginosa*.

## Author Contributions

YJ, WW, and SJ conceived and designed the experiments, and wrote the paper. YJ, MZ, FZ, QP, YW, QZ, and CL performed the experiments. YJ, WW, FB, ZC, and SJ analyzed the data.

## Conflict of Interest Statement

The authors declare that the research was conducted in the absence of any commercial or financial relationships that could be construed as a potential conflict of interest.

## Footnotes

1 www.pseudomonas.com

2 https://string-db.org/

## Supplementary Material

FIGURE S1 Growth curve of PAK and the ΔPA4916 mutant.

FIGURE S2 Role of NrtR in twitching motility or expression of *prpL* and *toxA* in *P. aeruginosa*. **(A)** Twitching motilities of the indicated strains were examined on 1% LB agar with a Δ*vfr* mutant serving as a control. The twitching zones were visualized with 0.1% crystal violet staining. **(B)** Relative mRNA levels of *prpL* and *toxA*. Total RNA of indicated strains was isolated and mRNA levels of *prpL* and *toxA* were determined by real time PCR with *rpsL* serving as an internal control. Data represents the mean ± standard deviation. ^∗∗^*p* < 0.01, ^∗∗∗^*p* < 0.001 by Student’s *t*-test.

FIGURE S3 PA4918 is not involved in the regulation of T3SS. **(A)** Relative mRNA levels of PA4918. Total RNA of indicated strains was isolated and mRNA levels of PA4918 were determined by real time PCR with *rpsL* serving as an internal control. Data represents the mean ± standard deviation. ^∗∗∗^*p* < 0.001 by Student’s *t*-test. **(B)** PAK containing the PA4918-20 operon driven by a *tac* promoter or the empty vector pMMB67EH were grown to an OD_600_ of 1.0 in LB with 1 mM IPTG with or without 5 mM EGTA. Proteins from equivalent bacterial cells of indicated strains were separated by SDS-PAGE and probed with an anti-ExoS antibody or an anti-RNA polymerase beta subunit antibody. S, supernatant; P, pellet.

FIGURE S4 Coomassie blue staining of purified CyaB_217-463_ and NadD2. **(B)** Purified NadD2 was not contaminated by ATPase. Equal amount ATP was incubated with or without 5 μg NadD2. After 30 min at 30°C, the ATP levels were measured using an ATP detection kit. Error bars represent standard deviations. ns, not significant, by Student’s *t-*test. **(C)** Purified NadD2 was not contaminated by phosphodiesterase. Equal amount cAMP was incubated with or without 5 μg NadD2. After 30 min at 30°C, the cAMP levels were measured using an ELISA kit. Error bars represent standard deviations. ns, not significant, by Student’s *t-*test.
