# Supplementary material for: NrtR Regulates the Type III Secretion System Through cAMP/Vfr Pathway in Pseudomonas aeruginosa
Source: Front Microbiol. 2019 Jan 30;10:85. doi: 10.3389/fmicb.2019.00085 (PMC6363681; doi:10.3389/fmicb.2019.00085)
Supplement: Supplementary file 1 [file Data_Sheet_1.PDF]

Supplementary Text

The pEX18Tc-Δ*cyaA* was constructed by cloning the 880 bp upstream and 963 bp downstream fragments of the *cyaA* gene into the *Eco*RI-*Hin*dIII sites of plasmid pEX18Tc. The pEX18Tc-Δ*cyaB* was constructed by cloning the 1019 bp upstream and 802 bp downstream fragments of the *cyaB* gene into the *Eco*RI-*Hin*dIII sites of plasmid pEX18Tc. The pEX18Tc-Δ*vfr* was constructed by cloning the 1090 bp upstream and 1064 bp downstream fragments of the *vfr* gene into the *Eco*RI-*Hin*dIII sites of plasmid pEX18Tc. The pEX18Tc-Δ*nadD2* was constructed by cloning the 816 bp upstream and 835 bp downstream fragments of the *nadD2* gene into the *Eco*RI-*Hin*dIII sites of plasmid pEX18Tc. The pEX18Tc-Δ*nadD2-nrtR* was constructed by cloning the 816 bp upstream and 1308 bp downstream fragments of the *nadD2-nrtR* operon into the *Hin*dIII site of plasmid pEX18Tc.
