# Supplementary figures and images for: NrtR Regulates the Type III Secretion System Through cAMP/Vfr Pathway in Pseudomonas aeruginosa
Source: Front Microbiol. 2019 Jan 30;10:85. doi: 10.3389/fmicb.2019.00085 (PMC6363681; doi:10.3389/fmicb.2019.00085)

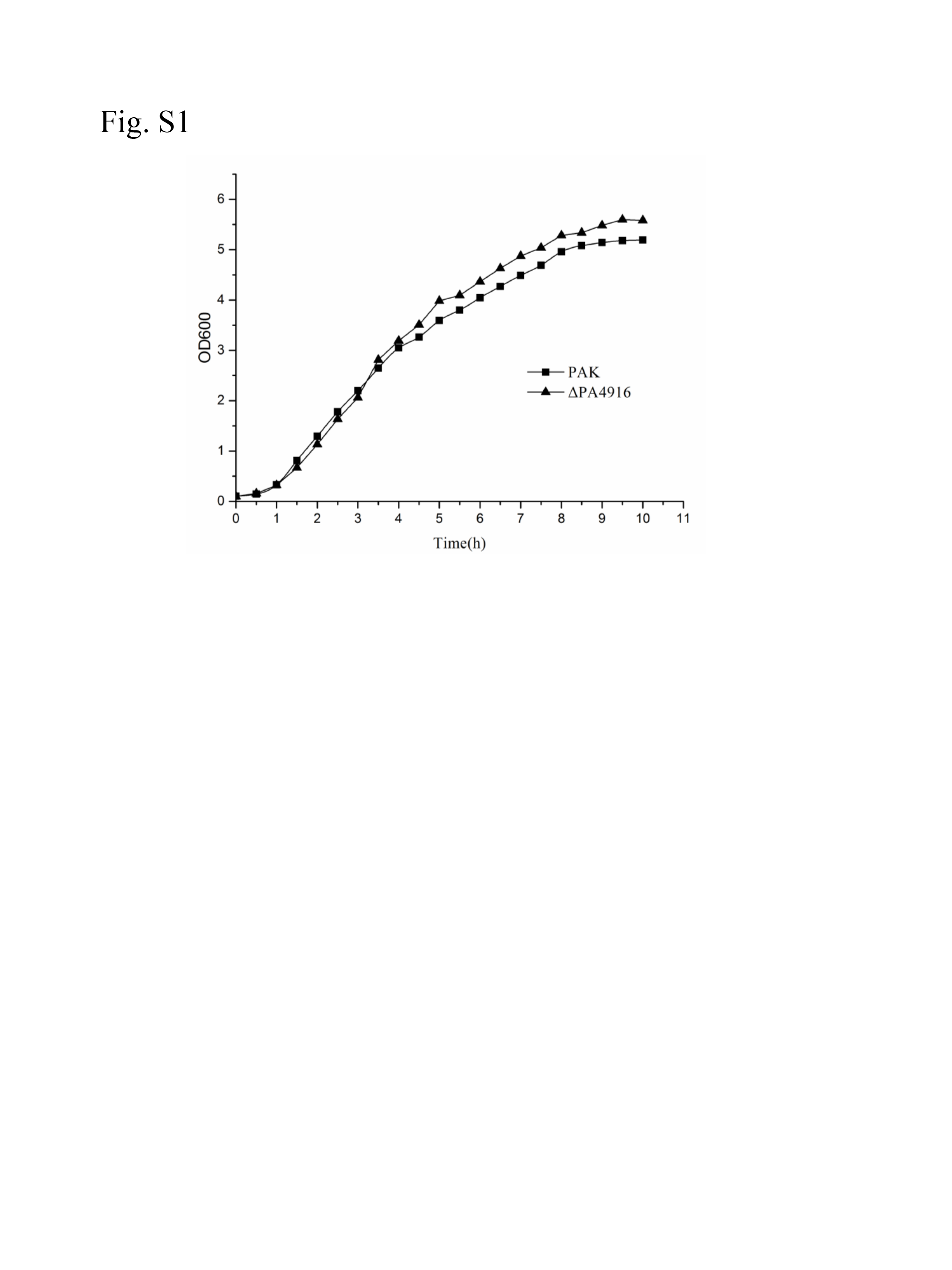

Supplement: FIGURE S1 — Growth curve of PAK and the ΔPA4916 mutant. [file Image_1.TIF]

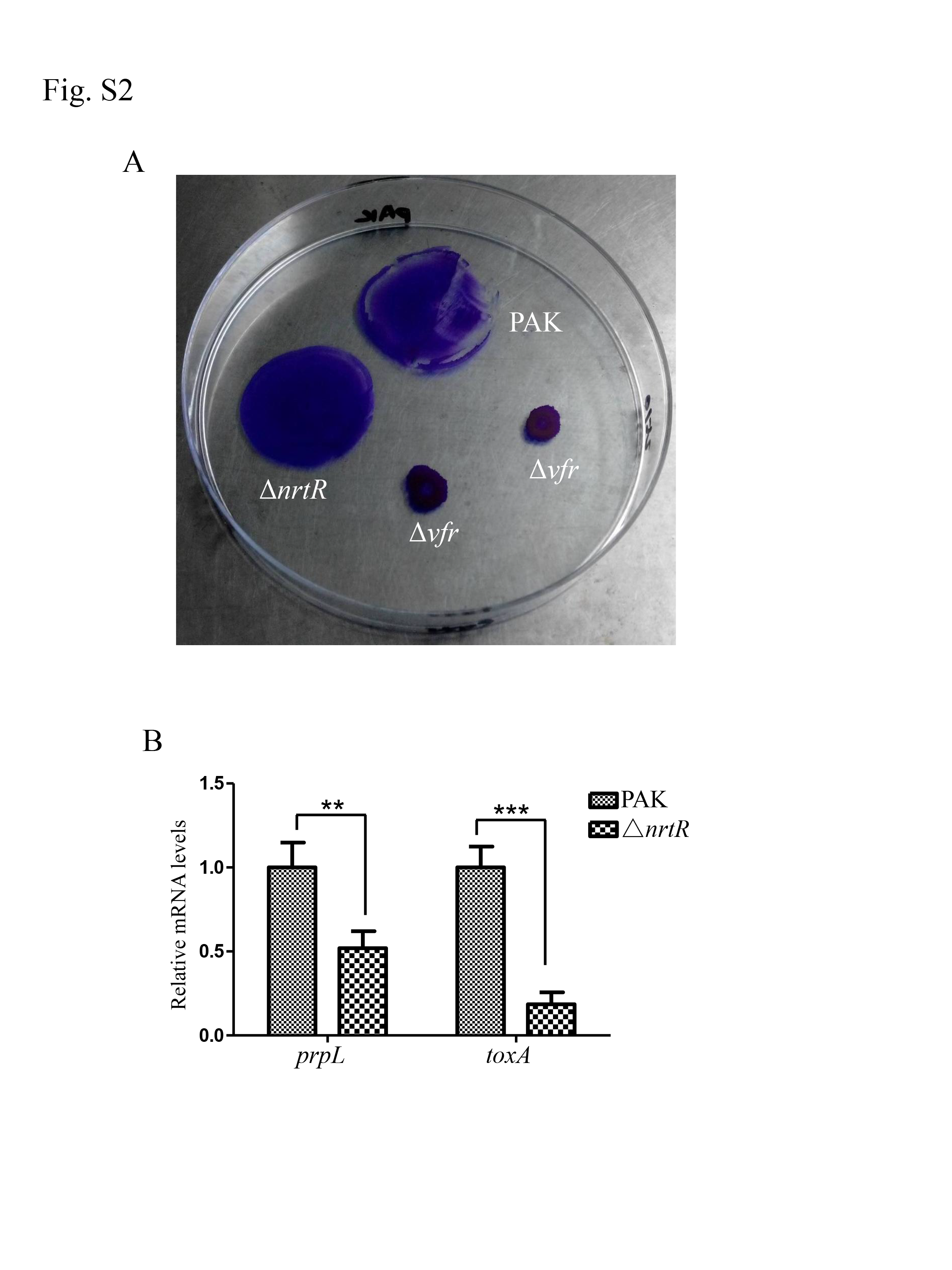

Supplement: FIGURE S2 — Role of NrtR in twitching motility or expression of prpL and toxA in P. aeruginosa. (A) Twitching motilities of the indicated strains were examined on 1% LB agar with a Δvfr mutant serving as a control. The twitching zones were visualized with 0.1% crystal violet staining. (B) Relative mRNA levels of prpL and toxA. Total RNA of indicated strains was isolated and mRNA levels of prpL and toxA were determined by real time PCR with rpsL serving as an internal control. Data represents the mean ± standard deviation. ∗∗p < 0.01, ∗∗∗p < 0.001 by Student’s t-test. [file Image_2.tif]

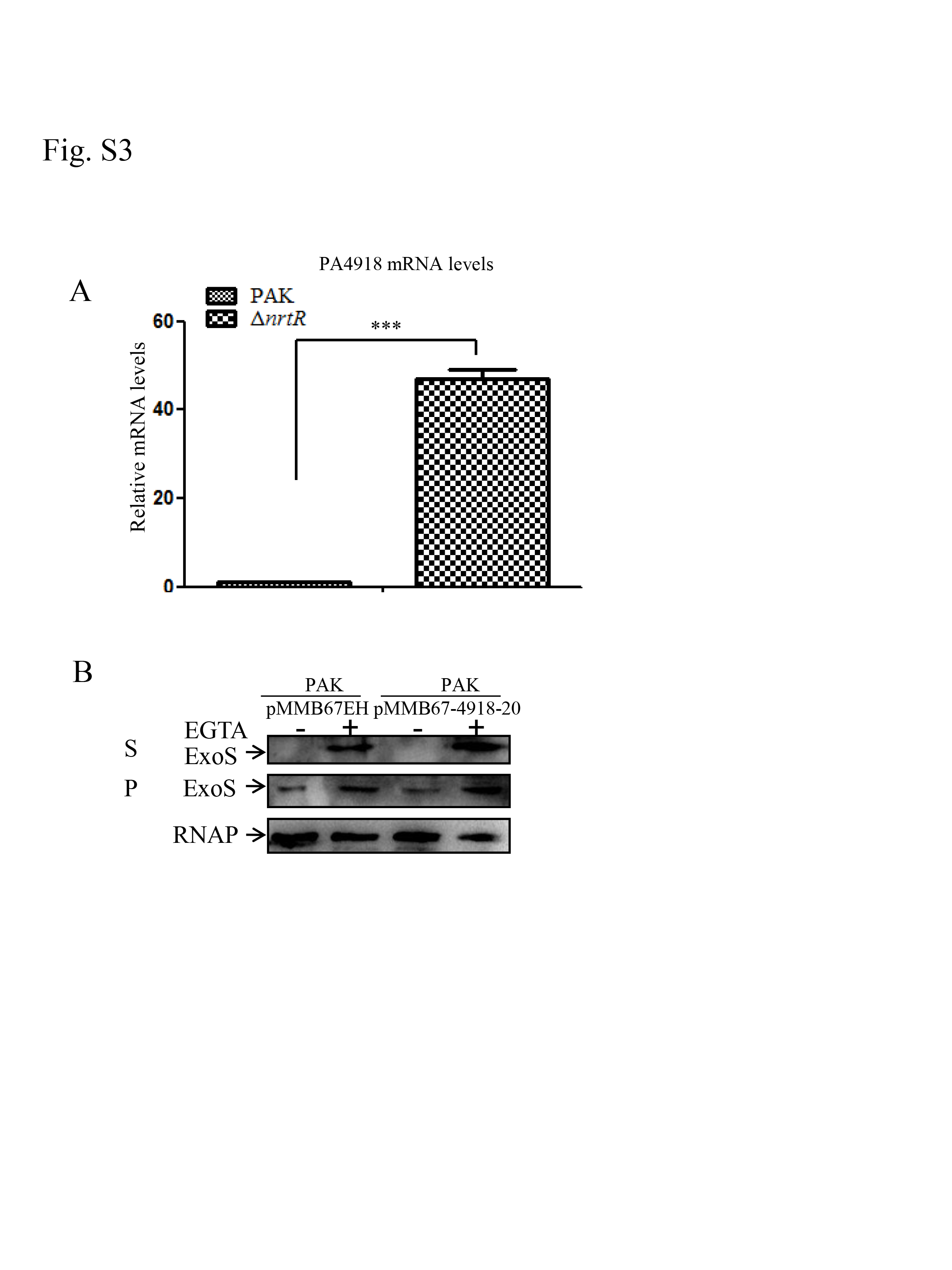

Supplement: FIGURE S3 — PA4918 is not involved in the regulation of T3SS. (A) Relative mRNA levels of PA4918. Total RNA of indicated strains was isolated and mRNA levels of PA4918 were determined by real time PCR with rpsL serving as an internal control. Data represents the mean ± standard deviation. ∗∗∗p < 0.001 by Student’s t-test. (B) PAK containing the PA4918-20 operon driven by a tac promoter or the empty vector pMMB67EH were grown to an OD600 of 1.0 in LB with 1 mM IPTG with or without 5 mM EGTA. Proteins from equivalent bacterial cells of indicated strains were separated by SDS-PAGE and probed with an anti-ExoS antibody or an anti-RNA polymerase beta subunit antibody. S, supernatant; P, pellet. [file Image_3.tif]

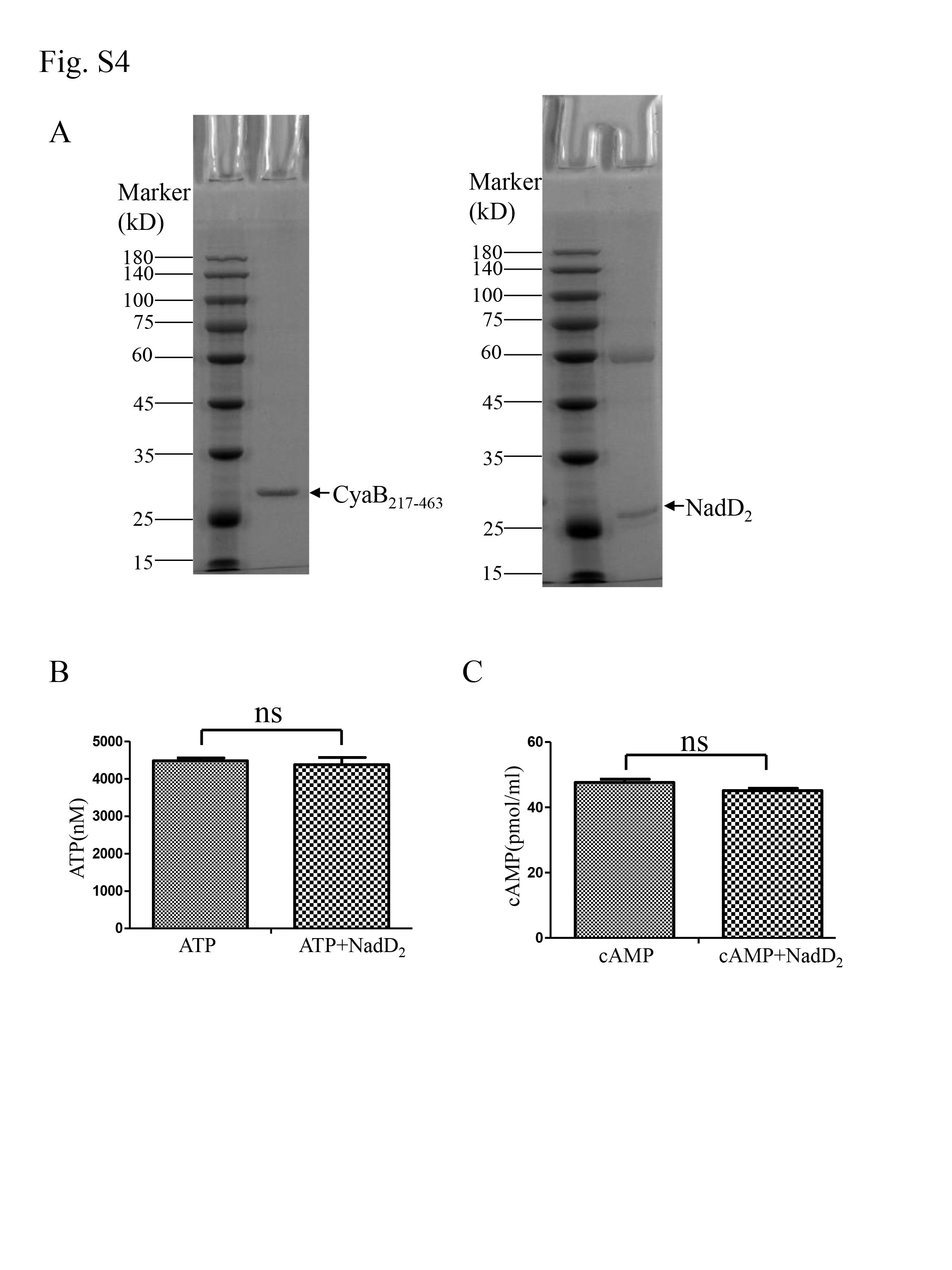

Supplement: FIGURE S4 — Coomassie blue staining of purified CyaB217-463 and NadD2. (B) Purified NadD2 was not contaminated by ATPase. Equal amount ATP was incubated with or without 5 μg NadD2. After 30 min at 30°C, the ATP levels were measured using an ATP detection kit. Error bars represent standard deviations. ns, not significant, by Student’s t-test. (C) Purified NadD2 was not contaminated by phosphodiesterase. Equal amount cAMP was incubated with or without 5 μg NadD2. After 30 min at 30°C, the cAMP levels were measured using an ELISA kit. Error bars represent standard deviations. ns, not significant, by Student’s t-test. [file Image_4.TIF]
